# Association Between METS-IR and Prehypertension or Hypertension Among Normoglycemia Subjects in Japan: A Retrospective Study

**DOI:** 10.3389/fendo.2022.851338

**Published:** 2022-03-18

**Authors:** Kai-Yue Han, Jianing Gu, Zhangsheng Wang, Jie Liu, Su Zou, Chen-Xi Yang, Dan Liu, Yingjia Xu

**Affiliations:** ^1^ Department of Cardiology, Shanghai Fifth People’s Hospital, Fudan University, Shanghai, China; ^2^ Department of Vascular and Endovascular Surgery, Chinese PLA General Hospital, Beijing, China

**Keywords:** metabolic score for insulin resistance (METS-IR), insulin resistance, prehypertension, hypertension, normoglycemia

## Abstract

**Aim:**

Our study aimed to investigate the association between the novel non–insulin‐based metabolic score for insulin resistance (METS-IR) index and pre-hypertension (HTN) or HTN in normoglycemia Japanese participants.

**Methods:**

The NAGALA medical examination program at Murakami Memorial Hospital in Gifu, Japan was found in 1994. 15,453 participants enrolled in this program from 2004 to 2015 was included in this retrospective study to explore the association between the METS-IR index and pre-HTN or HTN. Covariates included serum biomarkers and clinicodemographic characteristics. Logistic regression was applied to explore the association between METS-IR level and pre-HTN or HTN.

**Results:**

This study includes a total of 15453 participants. The prevalence rates of pre-HTN and HTN were 28.55% (4412/15453) and 6.23% (962/15453), respectively. Adjusted for confounding factors in the multivariable logistic regression analysis models, when METS-IR was used as a categorical variable, high METS-IR was significantly associated with both pre-HTN (adjusted odds ratio (OR) = 1.95, 95% confidence interval (CI): 1.61–2.36) and HTN (adjusted OR = 2.12, 95% CI: 1.44–3.11). When METS-IR was used as a continuous variable, each 1 unit increase in METS-IR was associated with a 7% increase in the prevalence of pre-HTN (adjusted OR = 1.07, 95% CI: 1.06–1.08) and with a 13% increase in the prevalence of HTN (adjusted OR = 1.13, 95% CI: 1.10–1.16). Stratified analyses indicated a positive correlation between METS-IR and pre-HTN or HTN in normoglycemia subjects with different characteristics.

**Conclusions:**

METS-IR levels are significantly associated with pre-HTN or HTN in normoglycemia individuals in Gifu, Japan. METS-IR may be used as a monitoring indicator for the development of HTN primary prevention and management strategies in the future, but it still needs more research to confirm.

## Introduction

Hypertension (HTN) and cardiovascular diseases (CVDs) caused by high blood pressure blood pressure have drawn great attentions in public health (1, 2). More recently, the prevalence of HTN has surged, resulting in the increase of blood pressure-related morbidity and death. Furthermore, the common concomitant status in prehypertensive and hypertensive individuals, such as abnormalities in glucose and lipid homeostasis, leads to a poorer long-term prognosis (3). In fact, dyslipidemia and impaired fasting glucose (IFG) were shown to be present in 41.9 percent and 40.7 percent of hypertensive individuals, respectively (4, 5). Aberrant glycolipid metabolism in hypertensive individuals considerably increases the risk of CVD in these patients. As a result, seeking for a better understanding of the glycolipid metabolism components in patients with HTN may help to alleviate the massive load of global disease.

Insulin resistance (IR), defined as the attenuation of insulin responsiveness in tissues, is a crucial mechanism in glycolipid metabolism (6). IR may be a substantially primary cause for CVD, according to current epidemiological and pathophysiology researches (6–8). The impact of IR on the pathophysiology of HTN has also been extensively studied (9). Up to now, the available the reference standard for evaluating the significance of IR is the hyperinsulinemic euglycemic clamp (HEC) (10). However, this approach of analyzing IR, is time-consuming, costly, and sophisticated, and it necessitates a large workforce. Consequently, it is unsuitable for routine clinical use. The development of non-insulin-based IR indicators has provided an easier and less expensive method to detect IR, especially in primary healthcare settings.

The metabolic score for insulin resistance (METS-IR) index is a recently developed index aimed to be a practical and efficient alternative biomarker of IR (11). The METS-IR index has a stronger correlation with the HEC than other non–insulin‐based IR indexes (11). However, there are only few studies on the association between the METS-IR index and blood pressure, with studies limited only to China and Mexico (12–15). Further, the enrolled populations in these studies were mainly those with a history of HTN and, therefore, were taking antihypertensive drugs. As such, the conclusions of these studies are easily affected by the use of medicine. Moreover, the association of METS-IR index with pre-HTN and HTN in different ethnic is unknown. Thus, we aim to study the association between METS-IR index and pre-HTN and HTN in Japanese normoglycemia individuals, using a relevant database (16).

## Methods

### Data Source

Our data source was the DATADRYAD database (http://www.Datadryad.org/). The information was obtained from the Dryad data package (Okamura, Takuro, et al., 2019), which can be downloaded for free for all researchers. Detailed citations were used to gain information on the study of Okamura, Takuro, et al. (dataset: 10.5061/dryad.8q0p192) (16). The following variables of enrolled participants in the database included: age, sex, waist circumference (WC), weight, body mass index (BMI), systolic blood pressure (SBP), diastolic blood pressure (DBP), alanine aminotransferase (ALT), fasting plasma glucose (FPG), total cholesterol (TC), aspartate transaminase (AST), γ-glutamyl transpeptidase (GGT), high-density lipoprotein cholesterol (HDL-C), smoking status, exercise, fatty liver, alcohol consumption, triglycerides (TG), hemoglobin A1c (HbA1c), obesity phenotype, obesity, visceral fat obesity, ethanol consumption, diabetes mellitus (DM), and follow-up duration.

### Study Population

A healthcare program aimed to identify chronic cardiovascular disorders and improve public health conditions was carried out at the hospital in Gifu, Japan. Data collected from the program were used to create the NAfld in the Gifu Area, Longitudinal Analysis (NAGALA) database (16). From 2004 to 2015, participants were given a test, with 60% of them receiving one or two exams per year. Based on exclusion criteria of (1) incomplete relevant records; (2) steatohepatitis or hepatitis B or C; (3) alcoholism (alcohol consumption over 60 g/day for men and 40 g/day for women); (4) oral intake of medicine; and (5) blood glucose level >6.1, a total of 15453 participants (8441 men and 7034 women) were finally enrolled in the program. The ethical approval was provided by Murakami Memorial Hospital’s ethical committee, and a written informed consent was obligately required for all participants.

### Data Collection and Measurements

The NAGALA database contained the medical history and lifestyle information of individuals based on a standardized questionnaire. Alcohol intake was measured by subdivision of alcohol and average weekly alcohol consumption over the previous month. Thus, there were four categories as followed: no or minimal drinker (40 g/week), light drinker (40–140 g/week), moderate drinker (140–280 g/week), and heavy drinker (>280 g/week) (17). Simultaneously, according to the habits of smoking, the participants can be allocated to three different groups: none, ex-smoker, or present smoker. The regular exercisers referred to those who participate in any type of sports more than one time per week regularly (18). Steatohepatitis should correspond to the definition of which diagnosed by abdominal ultrasonography (19). The definition of obesity was a body mass index (BMI) no less than 25 kg/m2 or higher (20–22). Visceral fat obesity refers to a waist circumference ≥90 cm in men or ≥80 cm in women (23). The METS-IR index was calculated as follows: Ln[(2 × fasting glucose (mg/dL))+fasting TG (mg/dL)] × BMI (kg/m^2^))/(Ln[high‐density lipoprotein cholesterol (mg/dL)]) (11). Pre-HTN and HTN were defined according to the Japanese Society of Hypertension Guidelines for the Management of Hypertension (JSH 2019) (24). The JSH 2019 cut-offs of office blood pressure for defining HTN are SBP ≥140 and/or DBP ≥90 mmHg; elevated blood pressure, SBP 130–139 and/or DBP 80–89 mmHg; and high normal blood pressure, SBP 120–129 mmHg and DBP <80 mmHg). Participants with an elevated blood pressure and with high normal blood pressure were collectively considered as having pre-HTN in the current study.

### Statistical Analysis

Data were categorized into continuous and categorical variables. Continuous variables were further divided into two types based on the normality of their distribution. Normally distributed continuous variables were presented as the mean ± standard deviation and compared between groups using the Student’s t-test. Meanwhile, non-normally distributed variables were presented as the median ± interquartile range (IQR) and compared between two groups using the Wilcoxon rank-sum test. Categorical variables were presented as percentages and compared using the chi-square test. The Kruskal-Wallis test or one-way analysis of variance were applied to assess the significance of differences in groups stratified by METS-IR index quartiles. The association between METS-IR index and pre-HTN or HTN was investigated using univariate and multivariate logistic regression analyses models. Univariate and multivariable logistic regression analyses were used to study the association between METS-IR index and pre-HTN or HTN. Three models were used: model 1, adjusted for sex and age; model 2, adjusted for age, sex, smoking status, alcohol consumption and WC; and model 3, adjusted for age, sex, smoking status, alcohol consumption, WC, ALT, AST, GGT, and TC levels.

In the models, the median value of the METS-IR index in each quadrant was utilized to perform linear trend tests. In addition, we used curve-fitting to assess the linear relationship between METS-IR and pre-HTN or HTN. To identify modifications and interactions, we used a stratified linear regression model and likelihood ratio test in subgroups of age (<65 or ≥65 years), sex (female or male), BMI (<25 kg/cm^2^or ≥25 kg/cm^2^), and WC (<90 cm in men, <80 cm in women vs. ≥90 cm in men and ≥80 cm in women). The software packages R (http://www.R-project.org, The R Foundation) and Free Statistics software versions 1.3 were used to perform all statistical analyses. Statistical differences were considered significant at P<0.05.

## Results

### Population

**Figure 1:**
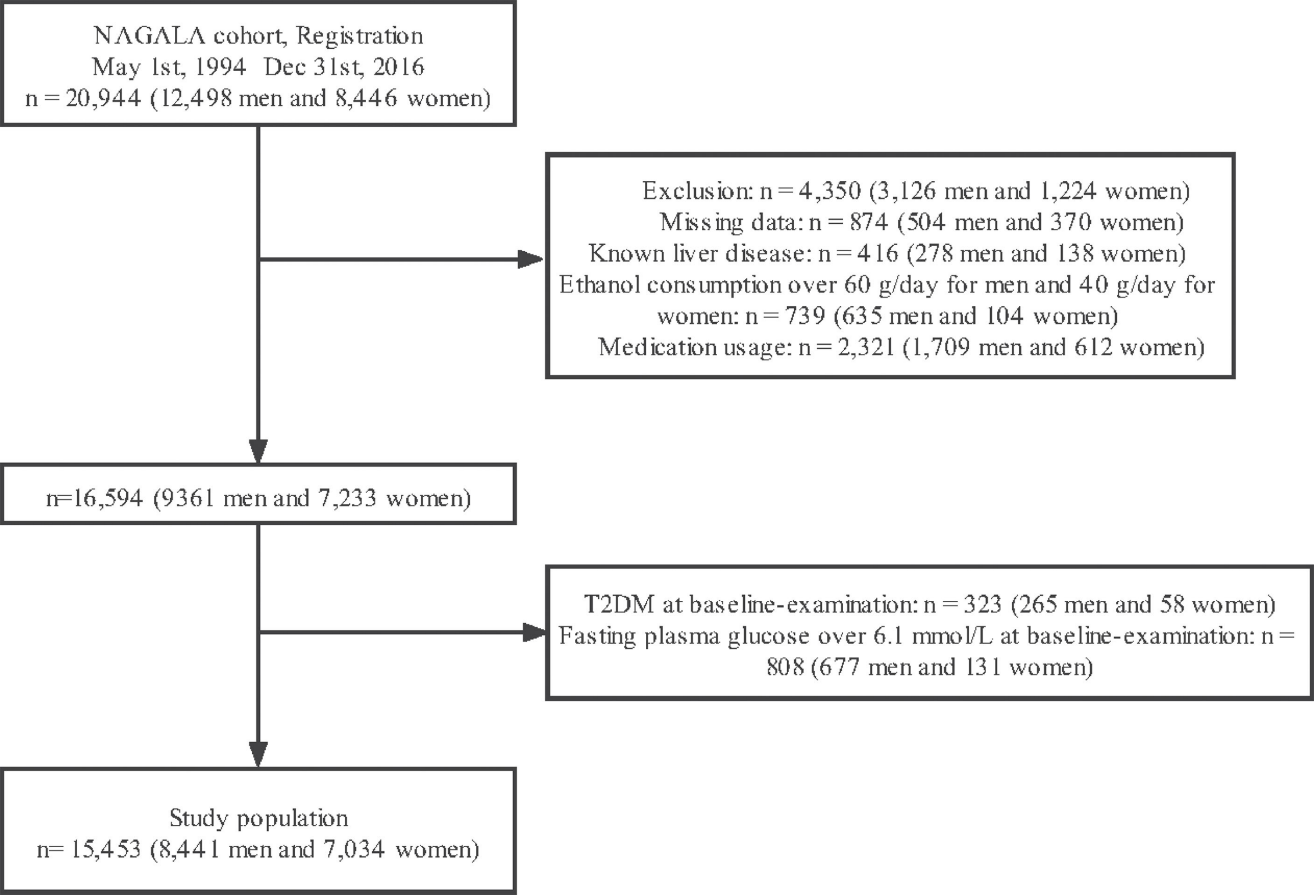
Flowchart of participant selection.

A total of 20,944 individuals were included in the NAGALA cohort. 4350 individuals were excluded owing to missing data (n=874), known liver disease (n=416), heavy drinking habits (n=739), and baseline medication consumption (n=2,321). At the baseline examination, 323 and 808 patients with T2DM and fasting blood glucose > 6.1 mmol/L, respectively, were further excluded. Consequently, 15,453 individuals were included in this study. The patient selection flowchart is shown in **Figure 1**.

### Baseline Characteristics

The mean participant age was 43.7 ± 8.9 years, and 7034 (45.5%) were men. The mean baseline METS-IR was 31.2 ± 6.5. The detailed characteristics of the population by METS-IR index quartiles are available in **Table 1**. METS-IR level was positively associated with several variables as follows: age, BMI, waist circumference, total cholesterol, TG, fasting blood glucose, aspartate aminotransferase, alanine aminotransferase, gamma glutamyl transferase, SBP, and DBP and inversely associated with HDL-C. Interestingly, the higher the METS-IR, the higher was the probability of high alcohol consumption and smoking. Meanwhile, women and those who exercised regularly had significantly lower METS-IR.

**Table 1:** Clinical characteristics of the study population according to METS-IR.

Variables	Total (n = 15453)	Q1 (n = 3863)	Q2 (n = 3863)	Q3 (n = 3863)	Q4 (n = 3864)	p value
Age, (years)	43.7 ± 8.9	41.6 ± 8.7	43.6 ± 8.9	45.0 ± 8.9	44.7 ± 8.6	<0.001
Male, n (%)	7034 (45.5)	809 (20.9)	1756 (45.5)	2679 (69.4)	3175 (82.2)	
BMI, (kg/m2)	22.1 ± 3.1	18.8 ± 1.3	21.0 ± 1.2	22.8 ± 1.4	25.9 ± 2.6	<0.001
WC, (cm)	76.5 ± 9.1	67.1 ± 5.0	73.2 ± 5.1	78.9 ± 5.1	86.6 ± 6.8	<0.001
WHtR	0.5 ± 0.0	0.4 ± 0.0	0.4 ± 0.0	0.5 ± 0.0	0.5 ± 0.0	<0.001
Alcohol consumption, n (%)						<0.001
None	11802 (76.4)	3290 (85.2)	2970 (76.9)	2764 (71.6)	2778 (71.9)	
Light	1754 (11.4)	304 (7.9)	462 (12)	491 (12.7)	497 (12.9)	
Moderate	1357 (8.8)	215 (5.6)	314 (8.1)	435 (11.3)	393 (10.2)	
Heavy	540 (3.5)	54 (1.4)	117 (3)	173 (4.5)	196 (5.1)	
Smoking status, n (%)						<0.001
Never	9027 (58.4)	3006 (77.8)	2482 (64.3)	1946 (50.4)	1593 (41.2)	
Past	2949 (19.1)	412 (10.7)	657 (17)	923 (23.9)	957 (24.8)	
Current	3477 (22.5)	445 (11.5)	724 (18.7)	994 (25.7)	1314 (34)	
Habit.of.exercise, n (%)						<0.001
No	12747 (82.5)	3212 (83.1)	3130 (81)	3124 (80.9)	3281 (84.9)	
Yes	2706 (17.5)	651 (16.9)	733 (19)	739 (19.1)	583 (15.1)	
HDL-c, (mg/dL)	56.5 ± 15.6	71.2 ± 14.6	60.3 ± 11.7	51.9 ± 9.9	42.7 ± 8.9	<0.001
TC, (mg/dL)	198.2 ± 33.4	192.0 ± 31.8	194.6 ± 32.8	199.5 ± 33.2	206.8 ± 33.9	<0.001
TG, (mg/dL)	65.0 (44.0, 99.0)	42.0 (31.0, 57.0)	55.0 (40.0, 74.0)	74.0 (54.0, 100.0)	117.0 (83.0, 164.0)	<0.001
HbA1, (%)	5.2 ± 0.3	5.1 ± 0.3	5.1 ± 0.3	5.2 ± 0.3	5.2 ± 0.3	<0.001
FPG (mg/dL)	93.0 ± 7.4	88.7 ± 6.9	91.8 ± 6.9	94.3 ± 6.7	97.1 ± 6.5	<0.001
ALT, (IU/L)	17.0 (13.0, 23.0)	14.0 (11.0, 17.0)	15.0 (12.0, 19.0)	18.0 (14.0, 23.0)	24.0 (18.0, 34.0)	<0.001
AST, (IU/L)	17.0 (14.0, 21.0)	16.0 (13.0, 19.0)	16.0 (13.0, 20.0)	17.0 (14.0, 21.0)	20.0 (16.0, 24.0)	<0.001
GGT, (IU/L)	15.0 (11.0, 22.0)	12.0 (10.0, 15.0)	13.0 (10.0, 17.0)	17.0 (12.0, 24.0)	22.0 (16.0, 33.0)	<0.001
SBP, (mmHg)	114.5 ± 15.0	106.4 ± 12.8	111.4 ± 13.2	116.9 ± 13.6	123.4 ± 14.6	<0.001
DBP, (mmHg)	71.6 ± 10.5	65.9 ± 8.9	69.3 ± 9.4	73.3 ± 9.6	77.9 ± 10.0	<0.001
METS-IR	31.2 ± 6.5	23.9 ± 1.7	28.2 ± 1.1	32.4 ± 1.4	40.1 ± 4.4	<0.001

Data were mean ± SD or median (IQR) for skewed variables or numbers (proportions) for categorical variables.

BMI, body mass index; WC, waist circumference; WHtR, waist‐to‐height ratio; HDL-c, high‐density lipoprotein cholesterol; TC, total cholesterol; TG, triglyceride; HbA1c, hemoglobin A1c; FPG, fasting plasma glucose; SBP, systolic blood pressure; DBP, diastolic blood pressure; ALT, alanine aminotransferase; ASL, aspartate aminotransferase; GGT, gamma glutamyl transferase; METS-IR, metabolic score for insulin resistance; Q1, Q2, Q3, and Q4 are quartiles of the metabolic score for insulin resistance(METS-IR).

### Univariate and Multivariate Analyses of Prehypertension and Hypertension

**Figure 2:**
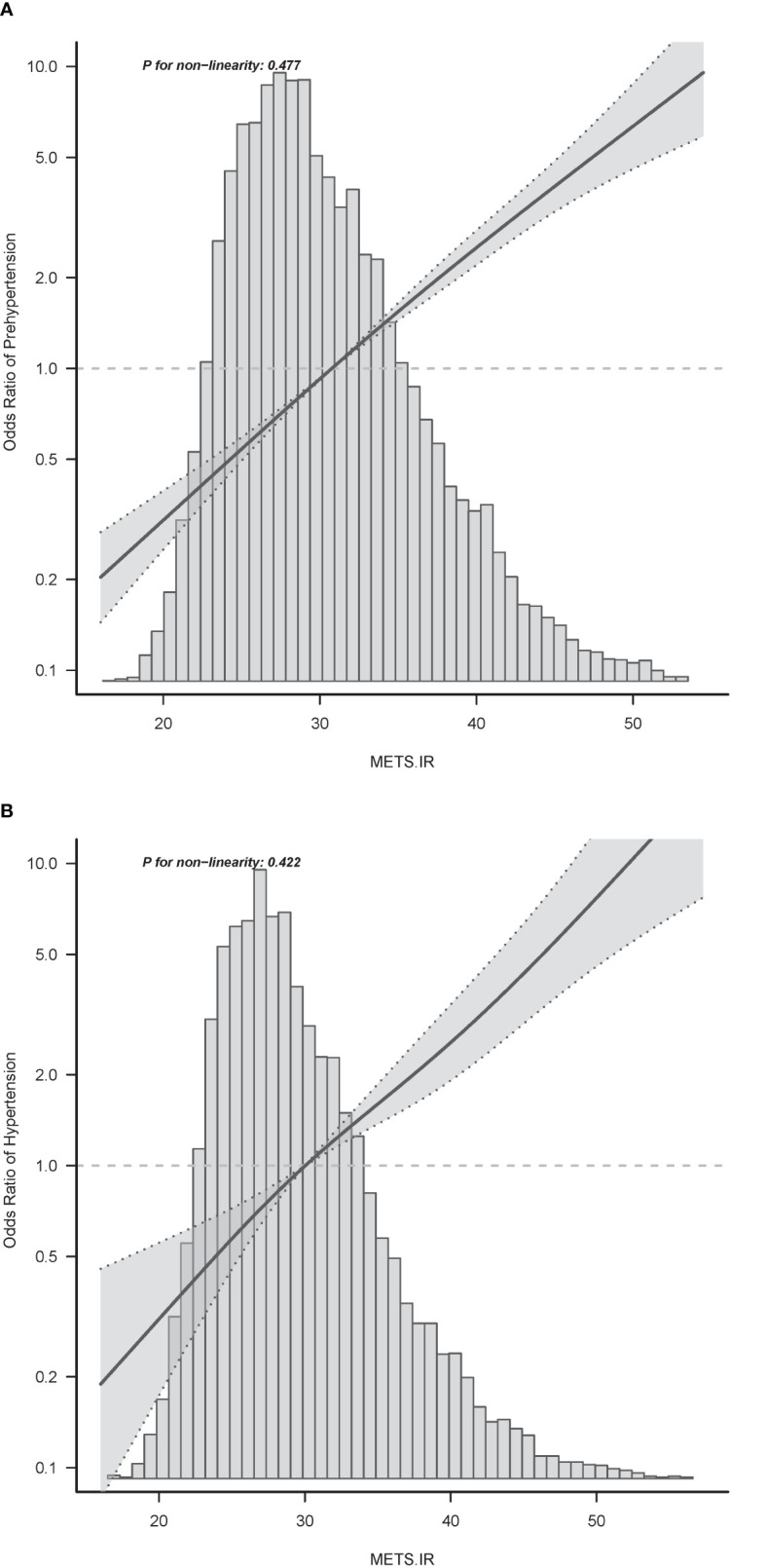
Associations between METS-IR index with prehypertension **(A)** or hypertension **(B)**. Odd ratios (ORs) were adjusted for age (continuous), sex (male or female), waist circumference (<90 or ≥90 in Men and <80(cm) ≥80 in Women), smoking status (never, past and current), alcohol consumption (none, light, moderate and heavy), total cholesterol (continuous) and triglyceride (continuous). Both P linearity, 0.001.

Age, sex, BMI, WC, smoking status, alcohol intake, GGT, ALT, AST, HDL-C, TC, TG, HbA1c, fasting plasma glucose, and METS-IR were significantly all associated with pre-HTN and HTN (**Table 2**). METS-IR as continuous variables was calculated in **Table 2**. There was a linear relationship between METS-IR index and pre-HTN or HTN (**Figure 2**). After adjusting for different confounders, METS-IR was inversely associated with pre-HTN or HTN in all three models (**Table 3**). The odds ratios (ORs) of METS-IR were consistently significant in all three models irrespective of whether METS-IR was analyzed as a continuous variable or quartile (OR range 1.07–1.95, p<0.05 for pre-HTN; OR range 1.07–2.12, p<0.05, except quartile 2(Q2) =0.692 for HTN).

**Table 2:** Results of univariate analysis of prehypertension and hypertension.

Variable	Prehypertension		Hypertension	
	OR (95%CI)	p value	OR (95%CI)	p value
Age, (years)	1.03 (1.03-1.04)	<0.001	1.07 (1.06-1.08)	<0.001
Sex, n (%)	2.84 (2.63-3.06)	<0.001	3.86 (3.31-4.50)	<0.001
BMI, (kg/m2)	1.3 (1.28-1.32)		1.44 (1.41~1.47)	<0.001
WC, (cm)	1.1 (1.09~1.10)	<0.001	1.14 (1.13~1.15)	<0.001
Smoking status,n (%)				
Never	ref		ref	
Past	1.94 (1.77~2.12)	<0.001	2.07 (1.76~2.44)	<0.001
Current	1.33 (1.22~1.45)	<0.001	1.31 (1.11~1.55)	0.001
Alcohol consumption				
None	ref		ref	
Light	1.54 (1.38~1.71)	<0.001	1.76 (1.44~2.15)	<0.001
Moderate	1.95 (1.73~2.20)	<0.001	2.91 (2.39~3.54)	<0.001
Heavy	2.56 (2.12~3.09)	<0.001	4.72 (3.61~6.15)	<0.001
ALT, (IU/L)	1.04 (1.03~1.04)	<0.001	1.04 (1.04~1.04)	<0.001
AST, (IU/L)	1.05 (1.05~1.06)	<0.001	1.05 (1.04~1.06)	<0.001
GGT, (IU/L)	1.03 (1.03~1.03)	<0.001	1.03 (1.03~1.03)	<0.001
HDL-c, (mg/dL)	0.98 (0.98~0.98)	<0.001	0.97 (0.96~0.97)	<0.001
TC, (mg/dL)	1.01 (1.01~1.01)	<0.001	1.01 (1.01~1.02)	<0.001
TG, (mg/dl)	1.01 (1.01~1.01)	<0.001	1.01 (1.01~1.01)	<0.001
HbA1c%	1.71 (1.53~1.92)	<0.001	2.25 (1.83~2.77)	<0.001
FPG, (mg/dl)	1.08 (1.08~1.09)	<0.001	1.12 (1.11~1.13)	<0.001
METS-IR	1.13 (1.12~1.13)	<0.001	1.18 (1.17~1.19)	<0.001

BMI, body mass index; WC, waist circumference; HDL-c, high‐density lipoprotein cholesterol; TC, total cholesterol; TG, triglyceride; HbA1c, hemoglobin A1c; FPG, fasting plasma glucose; ALT, alanine aminotransferase; ASL, aspartate aminotransferase; GGT, gamma glutamyl transferase; METS-IR, metabolic score for insulin resistance.

**Table 3:** Multivariable-adjust ORs and 95%CI of the METS-IR index quartiles associated with prehypertension and hypertension.

Variable	Unadjusted		Model 1		Model 2		Model 3	
	OR (95%CI)	p value	OR (95%CI)	p value	OR (95%CI)	p value	OR (95%CI)	p value
Prehypertension								
METS-IR	1.13 (1.12~1.13)	<0.001	1.11 (1.10~1.11)	<0.001	1.06 (1.05~1.07)	<0.001	1.07 (1.06~1.08)	<0.001
1st Quartile (≤26.29)	Ref		Ref		Ref		Ref	
2st Quartile (26.29-30.14)	1.86 (1.65~2.09)	<0.001	1.55 (1.37~1.75)	<0.001	1.16 (1.02~1.32)	0.025	1.18 (1.04~1.35)	0.012
3st Quartile (30.14-34.99)	3.35 (2.98~3.75)	<0.001	2.39 (2.12~2.70)	<0.001	1.38 (1.20~1.60)	<0.001	1.40 (1.21~1.63)	<0.001
4st Quartile (≥34.99)	7.04 (6.28~7.89)	<0.001	4.81 (4.24~5.44)	<0.001	2.02 (1.69~2.41)	<0.001	1.95 (1.61~2.36)	<0.001
p for trend	1.91 (1.85~1.98)	<0.001	1.69 (1.63~1.76)	<0.001	1.26 (1.19~1.34)	<0.001	1.24 (1.17~1.32)	<0.001
Hypertension								
METS-IR	1.18 (1.17~1.19)	<0.001	1.17 (1.15~1.18)	<0.001	1.13 (1.11~1.15)	<0.001	1.13 (1.10~1.16)	<0.001
1st Quartile (≤26.29)	Ref		Ref		Ref		Ref	
2st Quartile (26.29-30.14)	2.27 (1.67~3.10)	<0.001	1.77 (1.29~2.43)	<0.001	1.09 (0.78~1.50)	0.622	1.07 (0.77~1.48)	0.692
3st Quartile (30.14-34.99)	5.78 (4.35~7.69)	<0.001	3.72 (2.77~5.01)	<0.001	1.55 (1.12~2.14)	0.008	1.41 (1.02~1.97)	0.04
4st Quartile (≥34.99)	18.01 (13.72~23.64)	<0.001	10.92 (8.18~14.58)	<0.001	2.78 (1.94~3.99)	<0.001	2.12 (1.44~3.11)	<0.001
p for trend	2.73 (2.53~2.94)	<0.001	2.38 (2.19~2.58)	<0.001	1.5 (1.34~1.67)	<0.001	1.33 (1.18~1.50)	<0.001

Model 1 adjust for age and sex.

Model 2 adjust for Model 1+WC, Smoking status, Alcohol consumption.

Model 3 adjust for Model 1+Model 2+ALT, AST, GGT, TC, TG.

Ref, reference; METS-IR, metabolic score for insulin resistance; WC, waist circumference; ALT, alanine aminotransferase; ASL, aspartate aminotransferase; GGT, gamma glutamyl transferase.

When METS-IR was evaluated as a continuous variable, in the full variables adjusted model (model 3), the adjusted OR was 1.07 (95% CI: 1.06–1.08) for pre-HTN and was 1.13 (95% CI: 1.1–1.16) for HTN. When METS-IR was analyzed as quartiles, also in model 3, the adjusted OR for pre-HTN in Q2, Q3, and Q4 were 1.18 (95% CI: 1.04–1.35), 1.40 (95% CI: 1.21–1.63), and 1.95 (95% CI: 1.61–2.36), respectively, with quartile 1 as reference. In the same model and analysis, the adjusted ORs for HTN in Q2, 3, and 4 were 1.07 (95% CI: 0.77–1.48), 1.41 (95% CI: 1.02–1.97), 2.12 (95% CI: 1.44–3.11), and 1.33 (95% CI: 1.18–1.5), respectively, with quartile 1 as reference. Moreover, it was statistically significant in all models (**Table 3**, p for trend <0.001), indicating that METS-IR was inversely associated with pre-HTN and HTN.

### Subgroup Analyses by Adjusted Potential Effect Confounders

**Figure 3:**
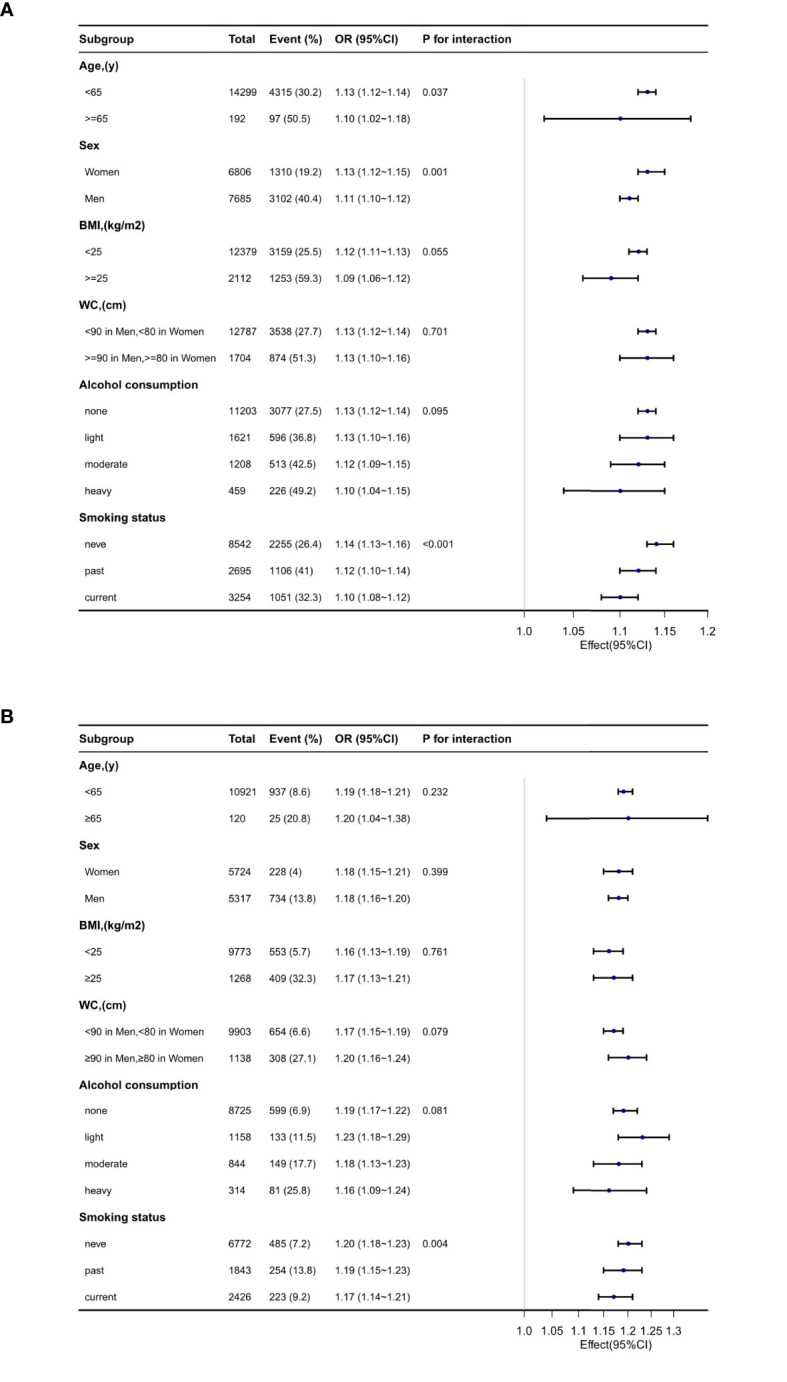
Subgroup analyses of the METS-IR and prehypertension **(A)** and hypertension **(B)**.

Subgroup analyses were performed to assess the impact of METS-IR (per 1 unit increment) on pre-HTN and HTN in distinct subgroups (**Figure 3**). The association between METS-IR and pre-HTN or HTN was coordinated in the subgroups as follows: in pre-HTN, age (<65 years vs. ≥65 years; P-interaction = 0.037), sex (female vs. male; P-interaction = 0.001), BMI (<24 kg/m^2^ vs. ≥24 kg/m^2^; P-interaction = 0.068), and WC (<90 cm in men, <80 cm in women vs. ≥90 cm in men and ≥80 cm in women; P-interaction = 0.701); in HTN, age (<65 years vs. ≥65 years; P-interaction = 0.232), sex (female vs. male; P-interaction = 0.399), BMI (<24 kg/m^2^ vs. ≥24 kg/m^2^; P-interaction = 0.966), and WC (<90 cm in men,<80 cm in women vs. ≥90 cm in men and ≥80 cm in women; P-interaction = 0.079).

## Discussion

After controlling for the variables in our population-based cross-sectional analysis, the results showed that the METS-IR, whether as a continuous or categorical variable, was positively and linearly associated with pre-HTN and HTN in normoglycemia subjects in Japan. The results were consistent in subgroups defined by age, sex, WC, and BMI (**Additional File 1: Tables S1**, **S2**).

Existing CVD risk assessment methods are unable to effectively assess the 10-year CVD risk in the prehypertensive populations (25); therefore, their CVD risk is unclear, highlighting an urgent need to develop more accurate and easy monitoring tools, particularly in primary care settings. Although HEC is the current gold standard for analyzing IR (26), it is mostly utilized only in research owing to its complexity. The most frequently used IR indicator in clinical and epidemiological studies is the homeostatic model assessment for insulin resistance (HOMA-IR), which is based on an insulin assay. However, its practical applicability is limited by its cost, particularly in less-developed areas (27). The METS-IR is a new substitute for IR that integrates traditional indicators (FPG, BMI, TG, and HDL) and shows good agreement with EHC and frequently sampled intravenous glucose tolerance (28).

The incidence of cardiovascular illnesses is higher in those with a BP of 120–129/80–84 mmHg and 130–139/85–89 mmHg than in those with a BP of 120/80 mmHg in Europe and the United States (29) and research in Japan (30, 31).Furthermore, the risk of HTN is also higher in those with a BP of 120–139/80–89 mmHg than those with a BP of 120/80 mmHg (32). Thus, we collectively defined those with an elevated blood pressure (SBP mmHg 130-139 and/or DBP 80–89 mmHg) and with high normal blood pressure (SBP 120–129 mmHg and DBP < 80 mmHg) based on JSH 2019 as having prehypertension in our study. Therefore, early detection and intervention for pre-HTN are important to avoid HTN. IR is a key aspect of pre-HTN and a necessary precursor to the early stages of the disease (3). However, up to now, there have been few studies on the relationship between BP and METS-IR. Fan et al. (14) compared the association of three alternative non-insulin-based IR surrogates with pre-HTN (defined SBP 120–139 and/or DBP 80–89 mmHg) in normoglycemia Chinese participants and found that only METS-IR, but not TG and triglycerides and high‐density lipoprotein cholesterol (TG/HDL-c), was associated with pre-HTN, irrespective of the categorization of WC. Interestingly, we found that METS-IR was also associated with pre-HTN in normoglycemia Japanese subjects and may be of value in the management of pre-HTN and the prevention of prediabetes in different ethnic groups.

Our findings in the HTN group were also similar to those by Fan et al. (covariates included age, sex, and smoking), with more robust results by including more covariates (age, sex, smoking status, WC, alcohol consumption, ALT, AST, GGT, and TC). Omar et al. (12) report that the METS-IR was strongly associated with arterial stiffness and could predict the development of arterial HTN when combined with the Framingham Hypertension Risk Prediction Model. In addition, the METS-IR index was superior to other previously validated non–insulin‐based IR measures, such as the TG index, TG/HDL-C index, and the HOMA‐IR index in their findings. As a new IR index, METS-IR was expected to become a predictor of incident HTN, and was a supplement to previously proven HTN risk prediction models.

Further, Liu et al. (13) indicated that, in normal-weight (BMI=18.5–23.9 kg/m2) Chinese adults but not in those with elevated BMI (≥24.0 kg/m2), METS‐IR was strongly associated with HTN. The formula for METS-IR includes the BMI. BMI is a well-established predictor of HTN, and it influences blood pressure through various processes, including IR. And obesity is accompanied by a rise in the prevalence of HTN. Conversely, actively losing weight can dramatically alleviate high blood pressure (33, 34). Consequently, according to BMI, using the METS-IR index might have a potential impact on monitoring and managing HTN. Our study found that the METS-IR index is associated with both pre-HTN and HTN in different BMI categories (<25.0 kg/m^2^ and ≥25.0 kg/m^2^) in the Japanese, which may be due to racial differences in BMI. Moreover, in our study, the mean BMI of HTN group was the highest, then that of pre-HTN, and the lowest was that of the normotensive group (**Additional File 1: Table S3**). Therefore, weight control is an essential preventive and therapeutic strategy for IR. Surprisingly, in subgroup analysis based on WC, the close relationship of METS-IR with pre-HTN and HTN remained significant, thus highlighting the need for more research into the mechanism of METS-IR independent of WC.

Pathophysiological data support an association among METSIR, arterial stiffness, and incident HTN. Hyperinsulinemia, hyperglycemia, dyslipidemia, HTN, and a pro-inflammatory state are all associated with IR, as are the consequences of disrupted insulin signaling at the endothelial cell level (endothelial cells and vascular smooth muscle cells). All of these factors lead to arterial stiffness and elevated arterial pressure (7, 35–38). As is known, the more activated the sympathetic nervous system is, as well as the more increased peripheral vascular resistance, and cardiac output, the higher the systemic blood pressure is, which are the most widely recognized theories linking IR and arterial HTN (39). Similarly, it is vital that the renin-angiotensin-aldosterone system would be fully activated by reduced insulin action, glucotoxicity, and MS, followed by risen tubular Na+ reabsorption and BP alterations (40). Endothelial dysfunction and decreased nitric oxide synthase activity are also caused by impaired insulin signaling, resulting in systemic vasoconstriction (41). We also found sexual dimorphism in METS-IR in both pre-HTN and HTN participants. Current evidence suggests that sex differences in IR may be related to the estrogen’s potential protective effect in the development of IR (42). Garbis et al. (43) provided insights into insulin dysregulation in young females with polycystic ovary syndrome, using serum proteomics. Some proteins associated with β-estradiol, lipid metabolism, inflammation, and vitamin D, which are biological traits of cardiovascular physiology, may partly explain the mechanism of sexual dimorphism (44). However, the exact underlying mechanism requires further investigation. This emphasizes the need for including gender as a biological variable in preclinical investigations to better understand the pathophysiology of cardiometabolic disorders.

There were also several limitations in this study. First, the cross-sectional study design restricted the capability to determine causation. Second, the HOMA-IR of IR was not determined, because insulin levels were seldom identified in large epidemiological studies. Third, because the study data were obtained from Japanese subjects, the generalizability of the findings to other ethnic groups is unknown. Fourth, because this research is based on a secondary analysis of previously published research data, the procedures performed during the medical consultation, such as taking blood pressure measurements, are not entirely clear. Finally, all variables showed striking significance, but this could be because the sample size was not adjusted. However, the data analysis was based on a large sample, thus making the findings relatively reliable.

In conclusion, the METS-IR level is associated with pre-HTN or HTN in normoglycemia individuals in Japan. METS-IR may be used as a monitoring indicator for the development of HTN primary prevention and management strategies in the future, but it still needs more research to confirm. 

## Data Availability Statement

The original contributions presented in the study are included in the article/**Supplementary Material**. Further inquiries can be directed to the corresponding author.

## Ethics Statement

The studies involving human participants were reviewed and approved by Murakami Memorial Hospital’s ethical committee. The patients/participants provided their written informed consent to participate in this study.

## Author Contributions

K-YH, JG, and YX designed the study. K-YH, JG, and ZW collected the data. K-YH, JG, SZ, and CXY analyzed the data. K-YH, JG, ZW, JL, SZ, CXY, and DL interpreted the result. K-YH wrote the first draft of the manuscript. YX contributed to the refinement of the manuscript. The final manuscript has been read and approved by the authors.

## Funding

This study was supported by Talent Development Plan funded by Shanghai Fifth People’s Hospital, Fudan University (No. 2020WYRCSG09).

## Conflict of Interest

The authors declare that the research was conducted in the absence of any commercial or financial relationships that could be construed as a potential conflict of interest.

## Publisher’s Note

All claims expressed in this article are solely those of the authors and do not necessarily represent those of their affiliated organizations, or those of the publisher, the editors and the reviewers. Any product that may be evaluated in this article, or claim that may be made by its manufacturer, is not guaranteed or endorsed by the publisher.
