# Supplementary material for: Association Between METS-IR and Prehypertension or Hypertension Among Normoglycemia Subjects in Japan: A Retrospective Study
Source: Front Endocrinol (Lausanne). 2022 Mar 18;13:851338. doi: 10.3389/fendo.2022.851338 (PMC8971288; doi:10.3389/fendo.2022.851338)
Supplement: Supplementary file 1 [file Table_1.docx]

**Additional file 1: table. S1 Subgroup analysis of METS-IR associated with preHTN.**

| **Subgroup** | **n.total** | **n.event%** | **crude.OR(95%CI)** | **crude.P_value** | **adj.OR(95%CI)** | **adj.P value** | **P.for.interaction** |
| --- | --- | --- | --- | --- | --- | --- | --- |
| **Age** |  |  |  |  |  |  |  |
| <65y | 14299 | 4315 (30.2) | 1.13 (1.12~1.13) | <0.001 | 1.13 (1.12~1.14) | <0.001 | 0.037 |
| ≥65y | 192 | 97 (50.5) | 1.05 (1~1.11) | 0.072 | 1.1 (1.02~1.18) | 0.016 |  |
| **Sex** |  |  |  |  |  |  |  |
| Women | 6806 | 1310 (19.2) | 1.13 (1.12~1.15) | <0.001 | 1.13 (1.12~1.15) | <0.001 | 0.001 |
| Men | 7685 | 3102 (40.4) | 1.09 (1.09~1.1) | <0.001 | 1.11 (1.1~1.12) | <0.001 |  |
| **BMI** |  |  |  |  |  |  |  |
| <25(kg/m2) | 12379 | 3159 (25.5) | 1.12 (1.11~1.13) | <0.001 | 1.12 (1.11~1.13) | <0.001 | 0.055 |
| ≥25(kg/m2) | 2112 | 1253 (59.3) | 1.08 (1.06~1.1) | <0.001 | 1.09 (1.06~1.12) | <0.001 |  |
| **WC** |  |  |  |  |  |  |  |
| <90(cm) in Men, <80(cm) in Women | 12787 | 3538 (27.7) | 1.13 (1.12~1.13) | <0.001 | 1.13 (1.12~1.14) | <0.001 | 0.701 |
| ≥90(cm) in Men,≥80(cm) in Women | 1704 | 874 (51.3) | 1.12 (1.1~1.14) | <0.001 | 1.13 (1.1~1.16) | <0.001 |  |
| **Alcohol consumption** |  |  |  |  |  |  |  |
| none | 11203 | 3077 (27.5) | 1.13 (1.12~1.14) | <0.001 | 1.13 (1.12~1.14) | <0.001 | 0.095 |
| light | 1621 | 596 (36.8) | 1.12 (1.1~1.14) | <0.001 | 1.13 (1.1~1.16) | <0.001 |  |
| moderate | 1208 | 513 (42.5) | 1.1 (1.08~1.12) | <0.001 | 1.12 (1.09~1.15) | <0.001 |  |
| heavy | 459 | 226 (49.2) | 1.1 (1.07~1.15) | <0.001 | 1.1 (1.04~1.15) | <0.001 |  |
| **Smoking status** |  |  |  |  |  |  |  |
| never | 8542 | 2255 (26.4) | 1.15 (1.14~1.16) | <0.001 | 1.14 (1.13~1.16) | <0.001 | <0.001 |
| past | 2695 | 1106 (41) | 1.11 (1.1~1.13) | <0.001 | 1.12 (1.1~1.14) | <0.001 |  |
| current | 3254 | 1051 (32.3) | 1.1 (1.09~1.11) | <0.001 | 1.1 (1.08~1.12) | <0.001 |  |

Data were mean ± SD or median (IQR) for skewed variables or numbers (proportions) for categorical variables.

Abbreviation: BMI, body mass index; WC, waist circumference;

**Additional file 1: table. S2 Subgroup analysis of METS-IR associated with HTN.**

| **Subgroup** | **n.total** | **n.event(%)** | **crude.OR(95%CI)** | **crude.P_value** | **adj.OR(95%CI)** | **adj.P_value** | **P.for.interaction** |
| --- | --- | --- | --- | --- | --- | --- | --- |
| **Age** |  |  |  |  |  |  |  |
| <65y | 10921 | 937 (8.6) | 1.18 (1.17~1.2) | <0.001 | 1.19 (1.18~1.21) | <0.001 | 0.232 |
| ≥65y | 120 | 25 (20.8) | 1.09 (1.01~1.19) | 0.037 | 1.2 (1.04~1.38) | 0.014 |  |
| **Sex** |  |  |  |  |  |  |  |
| Women | 5724 | 228 (4) | 1.19 (1.17~1.22) | <0.001 | 1.18 (1.15~1.21) | <0.001 | 0.399 |
| Men | 5317 | 734 (13.8) | 1.16 (1.14~1.17) | <0.001 | 1.18 (1.16~1.2) | <0.001 |  |
| **BMI** |  |  |  |  |  |  |  |
| <25(kg/m2) | 9773 | 553 (5.7) | 1.16 (1.14~1.18) | <0.001 | 1.16 (1.13~1.19) | <0.001 | 0.761 |
| ≥25(kg/m2) | 1268 | 409 (32.3) | 1.14 (1.11~1.17) | <0.001 | 1.17 (1.13~1.21) | <0.001 |  |
| **WC** |  |  |  |  |  |  |  |
| <90(cm) in Men, <80(cm) in Women | 9903 | 654 (6.6) | 1.16 (1.15~1.18) | <0.001 | 1.17 (1.15~1.19) | <0.001 | 0.079 |
| ≥90(cm) in Men,≥80(cm) in Women | 1138 | 308 (27.1) | 1.18 (1.16~1.21) | <0.001 | 1.2 (1.16~1.24) | <0.001 |  |
| **Alcohol** **consumption** |  |  |  |  |  |  |  |
| none | 8725 | 599 (6.9) | 1.18 (1.17~1.2) | <0.001 | 1.19 (1.17~1.22) | <0.001 | 0.081 |
| light | 1158 | 133 (11.5) | 1.21 (1.17~1.25) | <0.001 | 1.23 (1.18~1.29) | <0.001 |  |
| moderate | 844 | 149 (17.7) | 1.16 (1.12~1.19) | <0.001 | 1.18 (1.13~1.23) | <0.001 |  |
| heavy | 314 | 81 (25.8) | 1.14 (1.09~1.2) | <0.001 | 1.16 (1.09~1.24) | <0.001 |  |
| **Smoking status** |  |  |  |  |  |  |  |
| never | 6772 | 485 (7.2) | 1.21 (1.2~1.23) | <0.001 | 1.2 (1.18~1.23) | <0.001 | 0.004 |
| past | 1843 | 254 (13.8) | 1.16 (1.14~1.19) | <0.001 | 1.19 (1.15~1.23) | <0.001 |  |
| current | 2426 | 223 (9.2) | 1.16 (1.13~1.18) | <0.001 | 1.17 (1.14~1.21) | <0.001 |  |

Data were mean ± SD or median (IQR) for skewed variables or numbers (proportions) for categorical variables.

Abbreviation: BMI, body mass index; WC, waist circumference;

**Additional file 1: table.S3 The baseline characteristics of normotension, prehypertension, and hypertension.**

| **Variables** | **Normotension** | **Prehypertension** | **Hypertension** | ***p* value** |
| --- | --- | --- | --- | --- |
|  | **(n = 10079)** | **(n = 4412)** | **(n = 962)** |  |
| Sex, n (%) | 5496 (54.5) | 1310 (29.7) | 228 (23.7) | < 0.001 |
| Age,(years) | 42.6 ± 8.6 | 45.2 ± 9.2 | 48.2 ± 8.8 | < 0.001 |
| BMI,(kg/m2) | 21.3 ± 2.6 | 23.5 ± 3.2 | 24.8 ± 3.8 | < 0.001 |
| WC,(cm) | 74.0 ± 8.1 | 80.5 ± 8.7 | 84.0 ± 9.8 | < 0.001 |
| WHtR | 0.5 ± 0.0 | 0.5 ± 0.0 | 0.5 ± 0.1 | < 0.001 |
| Alcohol consumption, n (%) |  |  |  | < 0.001 |
| None | 8126 (80.6) | 3077 (69.7) | 599 (62.3) |  |
| Light | 1025 (10.2) | 596 (13.5) | 133 (13.8) |  |
| Moderate | 695 (6.9) | 513 (11.6) | 149 (15.5) |  |
| Heavy | 233 (2.3) | 226 (5.1) | 81 (8.4) |  |
| Smoking status, n (%) |  |  |  | < 0.001 |
| Never | 6287 (62.4) | 2255 (51.1) | 485 (50.4) |  |
| Past | 1589 (15.8) | 1106 (25.1) | 254 (26.4) |  |
| Current | 2203 (21.9) | 1051 (23.8) | 223 (23.2) |  |
| Habit.of.exercise, n (%) |  |  |  | 0.951 |
| No | 8318 (82.5) | 3639 (82.5) | 790 (82.1) |  |
| Yes | 1761 (17.5) | 773 (17.5) | 172 (17.9) |  |
| HDL-c,(mg/dL) | 58.3 ± 15.6 | 53.7 ± 15.2 | 51.3 ± 14.4 | < 0.001 |
| TC,(mg/dL) | 194.6 ± 32.7 | 203.9 ± 33.6 | 210.1 ± 33.7 | < 0.001 |
| TG,(mg/dl) | 58.0 (40.0, 86.0) | 79.5 (54.0, 120.0) | 96.0 (66.0, 144.0) | < 0.001 |
| HbA1c(%) | 5.2 ± 0.3 | 5.2 ± 0.3 | 5.2 ± 0.3 | < 0.001 |
| FPG,(mg/dl) | 91.5 ± 7.2 | 95.5 ± 7.0 | 97.1 ± 6.7 | < 0.001 |
| SBP,(mmHg) | 105.9 ± 8.7 | 127.0 ± 5.4 | 147.3 ± 11.2 | < 0.001 |
| DBP,(mmHg) | 66.0 ± 6.8 | 79.5 ± 5.3 | 93.9 ± 7.1 | < 0.001 |
| ALT,(IU/L) | 15.0 (12.0, 20.0) | 19.0 (14.0, 27.0) | 22.0 (16.0, 31.0) | < 0.001 |
| AST,(IU/L) | 17.0 (14.0, 20.0) | 19.0 (15.0, 23.0) | 19.0 (16.0, 24.0) | < 0.001 |
| GGT,(IU/L) | 14.0 (11.0, 19.0) | 19.0 (14.0, 28.0) | 22.0 (15.0, 34.0) | < 0.001 |
| METS-IR | 29.4 ± 5.5 | 33.9 ± 6.6 | 36.8 ± 7.7 | < 0.001 |

Data were mean ± SD or median (IQR) for skewed variables or numbers (proportions) for categorical variables;

Abbreviation: BMI, body mass index; WC, waist circumference; WHtR, waist‐to‐height ratio; HDL-c, high‐density lipoprotein cholesterol; TC, total cholesterol; TG, triglyceride;HbA1c, hemoglobin A1c; FPG, fasting plasma glucose; SBP, systolic blood pressure; DBP, diastolic blood pressure; ALT, alanine aminotransferase; ASL, aspartate aminotransferase; GGT, gammaglutamyltransferase; METS-IR, metabolic score for insulin resistance
